# Toxin–antitoxin systems and their role in disseminating and maintaining antimicrobial resistance

**DOI:** 10.1093/femsre/fux006

**Published:** 2017-03-16

**Authors:** Qiu E. Yang, Timothy R. Walsh

**Affiliations:** 1 Division of Infection and Immunity, Heath Park Hospital, Cardiff University, Cardiff CF14 4XN, UK

**Keywords:** toxin–antitoxins, addictive systems, antimicrobial resistance, persistence

## Abstract

Toxin–antitoxin systems (TAs) are ubiquitous among bacteria and play a crucial role in the dissemination and evolution of antibiotic resistance, such as maintaining multi-resistant plasmids and inducing persistence formation. Generally, activities of the toxins are neutralised by their conjugate antitoxins. In contrast, antitoxins are more liable to degrade under specific conditions such as stress, and free active toxins interfere with essential cellular processes including replication, translation and cell-wall synthesis. TAs have also been shown to be responsible for plasmid maintenance, stress management, bacterial persistence and biofilm formation. We discuss here the recent findings of these multifaceted TAs (type I–VI) and in particular examine the role of TAs in augmenting the dissemination and maintenance of multi-drug resistance in bacteria.

## Teaser

As antimicrobial resistance continues to escalate in Gram-negative bacteria, understanding the role of toxin–antitoxin systems in plasmid maintenance and inducing persistence becomes increasingly important.

## INTRODUCTION

Antibiotic resistance has been highlighted as one of the most pressing concern of 21th century. The rapid spread of ‘superbugs’, including Enterobacteriaceae with NDM-1 (New Delhi Metallo-beta-lactamase-1), KPC-2 (*Klebsiella pneumoniae* carbapenemase-2) and the most recent reported MCR-1 (mobile colistin resistance-1), has been described as a global crisis and an impending return to the pre-antibiotics *era* (Moellering 2010; Liu *et al*. 2015). To rationally combat antibiotic resistance, we require a better understanding of which factors influence the emergence and persistence of antibiotic resistant clones. Bacterial toxin–antitoxin systems (TAs), originally linked to plasmid maintenance systems (Ogura and Hiraga 1983), exert important activities in the context of bacterial resistance and persistence formation (Wen *et al*. 2014; Harms, Maisonneuve and Gerdes 2016; Patel 2016). TAs are small modules consisting of a stable toxin and its unstable cognate antitoxin. Antitoxins are more labile than toxins and readily degraded under stress conditions; this allow the toxins to exert their detrimental effects, promoting plasmid maintenance, slow growth and dormancy, which is rather linked with chromosomally encoded TAs (Page and Peti 2016). TAs are not essential for normal cell growth but are nonetheless widely present on bacterial plasmids and chromosomes. It has been hypothesised that TAs play a central role that is advantageous for cell survival in their natural habitat, such as switching into a dormant, drug-resistance state to withstand high levels of antibiotic stress (Page and Peti 2016). The toxins inhibit cell growth by targeting a variety of important cellular processes, including DNA replication, transcription and cell-wall synthesis, which are similar to antibiotic activities (Davies and Davies 2010; Chan, Balsa and Espinosa 2015). Because of their ubiquity and crucial intracellular targets, the study of bacterial toxins will help us understand their role in the dissemination and evolution of bacterial antibiotic resistance. In this review, we will provide a synopsis of TAs and in particular examine the role of type II TAs in augmenting the dissemination and maintenance of multidrug resistance in Gram-negative bacteria.

## TAs CLASSIFICATION

TAs are small genetic modules found on bacterial mobile genetic scaffolds like plasmids, as well as on bacterial chromosomes. The TA loci encode two-component systems that consist of a stable toxin whose overexpression either kills the bacterial cell or negates cell growth, and an unstable cognate antitoxin. As a result, when a plasmid encoding the TA is lost from a cell, the toxin is released from the existing TA complex and kills plasmid-free cells. In essence, this is an elegant model for perpetuating plasmid maintenance in bacterial population (Gerdes, Rasmussen and Molin 1986). This unique system is also called post-segregational killing. The first TA (*ccdAB*) identified was carried on a F-plasmid and was shown to play an important role in plasmid maintenance by coupling host cell division to plasmid proliferation (Ogura and Hiraga 1983). Since this initial discovery, a number of different TAs have been identified that are encoded on bacterial genomes. Based on their proteomic nature of their corresponding antitoxin, TAs are currently divided into six distinct classes (Table 1).

**Table 1.** The intracellular targets of TAs.

Targets of toxin	TA groups	Examples	Toxin	Antitoxin	Cellular process inhibited
Inner cell membranes	type I,V	*hok-sok, tisB-istR, ghoTS*	TisB, Hok, GhoT	TisA, Sok, GhoS	Cell membranes damage
Replication by DNA gyrase	type II	*ccdAB*, *parDE*	CcdB, ParE	CcdA, ParD	DNA replication
tRNA^fMet^	type II	*vapBC*	VapC	VapB	Translation
Ribosome-independent mRNA interferase	type II	*mazEF*	MazF	MazE	Translation
Ribosome-dependent mRNA interferase	type II	*relBE, higBA*	RelB, HigB	RelE, HigA	Translation
GltX:tRNA	type II	*hipBA*	HipA	HipB	Translation
Elongation factor EF-Tu	type II	*phd-doc*	Doc	Phd	Translation
Peptidoglycan precursors: UNAG	type II	*ω-ε-ζ*, *pezTA*	ζ, PezT	ε, PezA	Cell wall synthesis
Biofilm formation	type II,V	*mqsRA*, *ghoST*	MqsR, GhoT	MqsA, GhoS	Biofilm formation
mRNAs	type III	*toxIN,cptTN, tenpIN*	ToxN,CptN, TenpN	ToxI,CptI, TenpI	Growth arrest
Cytoskeletal protein MreB and FtsA	type IV	*yeeU-cbtA*	CbtA	YeeU	Cell morphology
Beta sliding clamp, protein DnaN	type VI	*socAB*	SocB	SocA	DNA elongation

### Type I TAs

All type I toxins are small hydrophobic proteins of approximately 60 amino acid and their gene expression is regulated by an antisense RNA transcribed from the toxin gene but in reverse orientation (Gerdes and Wagner 2007). Type I TAs are arranged either as overlapping, convergent transcribed genes pairs or as divergently transcribed gene pairs located apart. In the first case, the antitoxin is a *cis*-encoded antisense RNA (e.g. *hok-sok*, *bsrG-SR4*); in the latter case, it is a trans-encoded sRNA (e.g. *tisB-IstR1*, *shoB-ohsC*) (Brantl 2012). The first and best studied type I system is *hok-sok* (host killing, *hok*, and suppressor of killing, *sok*), which was first discovered on plasmid R1 from *Escherichia coli* (Gerdes, Rasmussen and Molin 1986; Thisted and Gerdes 1992). Later, other type I TAs were found in *E. coli* such as *ldr-rdl*, *tisB-istR1*, *ibs-sib*, *shoB-ohsC* and *symER* (Fozo 2012; Kawano 2012; Wagner and Unoson 2012).

All the toxins of type I TAs have an identical secondary structure consisting of one α-helical structure and are predicted to be localised in the inner membrane, and thus to induce pores into the bacterial cell membranes, resulting in inhibition of ATP synthesis (Fozo, Hemm and Storz 2008). Consequently, replication, transcription and translation maybe inhibited, leading to cellular death (Unoson and Wagner 2008). For instance, TisB produces clusters of narrow anion-selective pores in lipid bilayers that significantly disturbs the cytoplasmic membrane (Wagner and Unoson 2012). Many toxins are not bacteriocidal, but interfere with phage propagation, modulate the cell membrane or prevent mature particle formation, and in some cases, only overexpression of toxin genes shows a toxic effect (Yamaguchi and Inouye 2011).

### Type II TAs

Type II TAs have been most extensively studied among all TAs, and the huge number of type II TAs varies greatly from different bacterial species, even among the same species. Hitherto, 12 subgroups of type II TAs have been identified based on toxin amino sequence homology (Leplae *et al*. 2011), including *mazEF* (Aizenman, Engelberg-Kulka and Glaser 1996), *relEB* (Takagi *et al*. 2005), *yefM-yoeB* (Kamada and Hanaoka 2005), *ω-ε-ζ* (Zielenkiewicz and Ceglowski 2005) and *mqsRA* (Gonzalez Barrios *et al*. 2005; Brown *et al*. 2009). In type II systems, the antitoxin is a small, unstable protein that sequesters the toxin through protein complex formation. The expression of the two genes is regulated at the level of transcription by the TA complex that involves binding palindromic sequence at the promoter region. Therefore, as the concentration of the TA complex in the cell is reduced as a result of antitoxin degradation, the TA operon expression is suppressed to produce more toxin and antitoxin, and thus the type II system is also termed the ‘addiction module’ (Yarmolinsky 1995). In most cases, the antitoxin genes are located upstream of their cognate toxin genes so that the antitoxins appear to have an advantage for their production over their cognate toxins. Conversely, there are many exceptions of this genetic arrangement, such as *higBA* in which the toxin genes *higB* is located upstream of its cognate antitoxin genes, *higB* (Yamaguchi, Park and Inouye 2009; Christensen-Dalsgaard, Jørgensen and Gerdes 2010).

### Type III TAs

The *toxIN_Pa_* was first identified on a plasmid from *Erwinia carotovora* subspecies atrosepticum (*Pectobacterium atrosepticum*) as an example of the novel type III protein–RNA TAs (Fineran *et al*. 2009). The *toxIN_Pa_* locus consists of a toxin ToxN*_Pa_* inhibiting bacterial growth and RNA antisense ToxI*_Pa_* counteracting the toxicity. The arrangements of type III TAs are unusual, as a toxin gene is preceded by a short palindromic repeat, which separates the toxin from its small RNA antitoxin, composed of several repeats of short nucleotide sequences. The short palindromic repeat acts as a transcriptional terminator, regulating the relative levels of antitoxin and toxin transcript. For example, *toxIN_Bt_* located on pAW63 from *Bacillus thuringiensis* encodes a toxic protein ToxN_Bt_ and a antitoxin ToxI_Bt_ containing 2.9 tandem repeats of a 34-nucleotide sequence (Short, Monson and Salmond 2015; Goeders *et al*. 2016). Currently, type III TAs are divided into three subgroups sharing the same genetic organisation, namely *toxIN*, *cptIN* (for *Coprococcus* type III inhibitor-toxiN) and *tenpIN* (for type III ENdogenous to *Photorhabdus* inhibitor-toxiN) (Blower *et al*. 2012; Goeders *et al*. 2016). Though these subgroups were identified by shared identity with ToxN, their cognates diverge between and within the subtypes, such as the number of repeats and the length of repeats (Blower *et al*. 2012; Goeders *et al*. 2016). All type III toxins discovered so far serve as endoRNase that cleave mRNAs in adenine-rich regions, whose activities inhibit by forming RNA pseudoknot–toxin complex.

### Type IV TAs

The *yeeU-cbtA* has been proposed for the new type IV TAs in which the protein antitoxin interferes with binding of the toxin to its target rather than inhibiting the toxin via direct TA binding (Masuda *et al*. 2012). Unlike most toxins targeting the macromolecular biosynthesis, CbtA is the first toxin of the TAs that affects cellular morphology (Tan, Awano and Inouye 2011). CbtA binds and inhibits the polymerisation of bacterial cytoskeletal proteins, MreB and FtsZ. The antitoxin, YeeU, suppresses the CbtA toxicity by stabilising the CbtA target proteins rather than by directly interacting with CbtA to suppress its toxicity (Masuda *et al*. 2012). Specifically, YeeU directly binds to both MreB and FtsZ and enhances the bundling of their filaments *in vitro*. Notably, this is a unique feature of the *yeeU-cbtA* system, distinguishing it from all the other TAs in that CbtA and YeeU does not form a complex. Nevertheless, YeeU is able to neutralise CbtA toxicity. Thus, the *yeeU-cbtA* constitutes a new type of TA.

### Type V TAs

The *ghoTS* is a new type of TA, where GhoS (ghost cell suppressor) is the first known antitoxin to neutralise the toxicity of GhoT ghost cell toxin, by specifically cleaving its mRNA (Wang *et al*. 2012). Compared to the high overlapping catalytic sites of CRISPR-associated-2 protein SSO1404 structures, Wang *et al*. (2012) suggested that the antitoxin, GhoS, is a sequence-specific endoRNase that cleaves *ghoT* transcription and thereby prevents GhoT translation. GhoT is a membrane-damaging protein, and its production can lyse the cell membrane and change its morphologies. Ultimately, this causes the formation of ghost cells, a group of dead or dying cells in which cell outline is still visible but the cytoplasmic area is transparent (Wang *et al*. 2012). GhoT has also been shown to contribute to biofilm formation—after the deletion of its repressor GhoS, the formation of biofilm and cell motility increased by approximately 6- and 2-fold, respectively (Wang *et al*. 2012).

### Type VI TAs

In contrast to typical TAs, in which toxicity of the toxin is neutralised by the antitoxin, *socB* is unique and constitutively controlled by the protease CIpXP, while its cognate *socA* acts as a proteolytic adaptor, promoting the degradation of SocB by CIpXP. SocB identified in *Caulobacter crescentus* has been proposed to inhibit DNA replication through direct interaction with DnaN, a ring-shaped protein that encodes a central component for DNA elongation (Markovski and Wickner 2013). This interaction disrupts the association of DnaN and Pol III and other DnaN-binding proteins, resulting in the collapse of the DNA replication forks. It also has been shown that this DNA damage can cause the accumulation of SocB, suggesting that it may play a regulatory role in the induction of the RecA-mediated SOS response (Aakre *et al*. 2013; Markovski and Wickner 2013; Page and Peti 2016). Therefore, the *socA-socB* system may play important roles in promoting *Caulobacter* adaptation to varying environmental conditions by preventing DNA replication.

## THE CELLULAR TARGETS OF TAs

**Figure 1.**
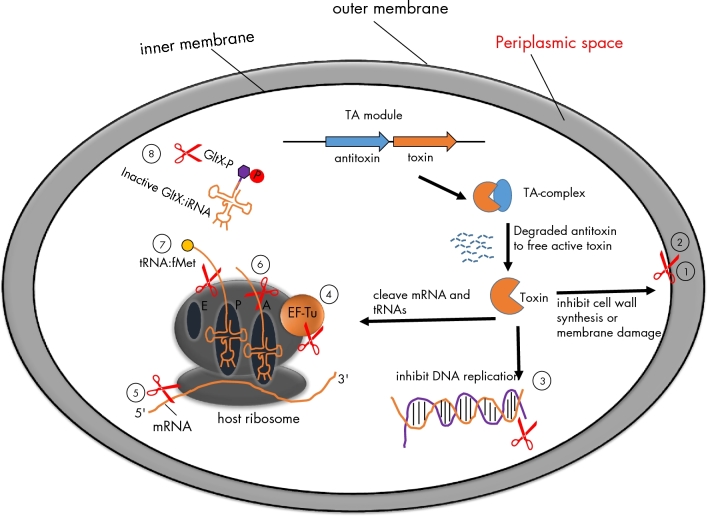
The intracellular targets of TAloci. TA loci usually encode two genes: one is a stable toxin and the other one is an unstable antitoxin. The antitoxins sequester the toxins but are subjected to proteolytic degradation by cellular proteases (Lon or ClpXP) under stress condition. Consequently, free active toxins alter cellular processes including DNA replication, translation or cell-wall synthesis, which ultimately results in slow growth or the formation of highly drug-tolerant persisters. TAs examples for the cellular targets are given below. (1) Zeta toxin inhibits cell-wall synthesis by specific phosphorylation of peptidoglycan precursor UNAG. (2) TisB, HokB and GhoT: the products of TisB and HokB can decrease the level of membrane potential motive force (pmf) and ATP by inserting into cytoplasmic membrane, while protein GhoT can lyse cell membrane and change cell morphologies. (3) CcdB and ParE inhibit DNA replication by poison DNA gyrase. (4) Doc inhibits translation by phosphoralation of elongation factor Tu (EF-Tu). (5–7) MazF, RelB and VapC inhibit translation by cleavage of mRNAs like single-stranded mRNA, A-site on ribosome and initiator tRNA^fMet^, respectively. (8) HipA inhibits translation by phosphoration of GltX. tRNA:fMet indicates initiator tRNA at P site carried formyl methionine; ‘p’ indicates phosphorylation.

In last decade, an increasing number of cellular targets for TAs have been elucidated, and most of them are involved in many essential bacterial processes such as DNA replication, RNA transcription and protein translational modification as shown in Table 1 and Fig. 1. Interestingly, TAs share many cellular targets with that of the antibiotics. For instance, zeta toxin phosphorylates the essential nucleotide sugar UDP-N-acetylglucosamine (UNAG), and leads to the inhibition of cell-wall synthesis, much like the activity of penicillin, that inhibits the formation of peptidoglycan across-links in the bacterial cell wall or glycopeptides that bind cell-wall precursors (Kohanski, Dwyer and Collins 2010). Another example are DNA gyrases that can induce and relax supercoils during DNA replication yet are the target of toxins CcdB and ParE, as well as quinolone antibiotics that disrupt DNA replication by binding to DNA-gyrase complexes (Kohanski, Dwyer and Collins 2010). Due to their remarkable similarity in cellular targets between TAs and antibiotics, TAs may provide novel insights into the discovery and development of new antimicrobials.

### Targeting cell-wall synthesis: Zeta toxin

The epsilon zetas were originally discovered as plasmid maintenance modules on a 29-kb low-copy plasmid, pSM19035, isolated from *Streptococcus pyogenes* (Zielenkiewicz and Ceglowski 2005). pSM19035 stability is conferred by two regions (*segA* and *segB*), and their corresponding products, SegA and SegB, control the plasmid partitioning (Ceglowski *et al*. 1993; Ana *et al*. 2000). The *segB* gene complex consists of four genes (δ and *ω-ε-ζ*), ensuring a ‘better-than-random’ plasmid segregation. The gene δ shares a significant homology to ATPases involved in active plasmid partitioning, but stabilisation function is dependent on the *ω-ε-ζ* operon. Therefore, among TAs, the organisation of the *ω-ε-ζ* operon is unique. The first three-component operon with the *ε* and *ζ* genes encodes an antitoxin and toxin, respectively, and the transcription of this operon is controlled by the additional gene ω (Ana *et al*. 2000; Zielenkiewicz and Ceglowski 2005). It has been show that the product of ω binds to the promoter of the entire operon as a dimer, and in the absence of ω repression, the intensity of transcription from ω is increased about 40-fold (Ana *et al*. 2000). Plasmid-encoded epsilon-zeta TAs enhance plasmid maintenance by killing the plasmid-free daughter cells (Zielenkiewicz and Ceglowski 2005), whereas chromosomally encoded epsilon-zeta TA family (*pezAT* for pneumococcal epsilon-zeta) identified from *S. pneumoniae* kills bacteria through the inhibition of cell-wall synthesis. More recently, Mutschler and Meinhart (2011) showed that toxin PezT inhibits the bacteria cell-wall synthesis by phosphorylating the UNAG into a toxic module UNAG-3-phosphate (UNAG-3P). UNAG-3P accumulates and competitively inhibits MurA, which is the essential enzyme for peptidoglycan synthesis (Barreteau *et al*. 2008), thereby freeing PezT toxin that poisons bacteria through inhibition of the cell-wall formation, causing the cells to lyse (Mutschler and Meinhart 2011; Mutschler *et al*. 2011).

The zeta toxin systems have been identified as highly abundant modules in multi-resistance plasmids and chromosomes of various Gram-positive pathogens, including *S. pyogenes* (Zielenkiewicz and Ceglowski 2005) and *S. pneumoniae* (Khoo *et al*. 2007). It has long been thought that epsilon-zeta systems only can be found in Gram-positive bacteria; however, a novel zeta homolog has been first identified from the Gram-negative bacterium *Escherichia coli* (Rocker and Meinhart 2015). This zeta toxin, designated EzeT for *E. coli* zeta toxin, is located in 3.4-kb islet, consisting of two domains featuring EzeT toxin and epsilon-like antitoxin within a single polypeptide chain. Similar to the toxin PzeT, the C-terminal domain of EzeT containing all catalytic motifs of UNAG kinases is capable of phosphorylating UNAG *in vitro* (Mutschler *et al*. 2011; Rocker and Meinhart 2015). In contrast to conventional type II TAs, N-terminal domain of EzeT contains an antitoxin; thus, EzeT is an authentic zeta-like UNAG kinase and is also the first auto-inhibited TA system, since it can be inhibited by its own N-terminal *cis*-acting antitoxin domain (Rocker and Meinhart 2015).

### Targeting tRNAs: VapC and HipA

PIN (N-terminus of the pilin biogenesis protein PiIT) domains are small protein domains consisting of 130 amino acid in length, and serve as ribonuclease enzymes that cleave single-stranded RNA in a sequence-dependent manner (Arcus *et al*. 2011). The TA module *vapBC* (virulence-associated protein) is associated with PIN-domain proteins. The *vap*BC (at that time called *vag*CD) locus derived from virulence plasmid of *Salmonella* Dublin strain G19 was proposed to prevent plasmid loss under nutrient-limiting conditions (Pullinger and Lax 1992). VapC is the PIN-domain ribonuclease, co-expressed with cognate inhibitor VapB that forms a novel PIN-domain–inhibitor complex (Bunker *et al*. 2008; Arcus *et al*. 2011). *vapBC* are surprisingly abundant; for example, the genome of *Mycobacterium tuberculosis* encodes 47 different *vapBC* homologs (Ahidjo *et al*. 2011). The transcription of *vapBC* operon is regulated by the DNA promoter, via the N-terminal ribbon-helix-helix domain of antitoxin VapB. The proteolytic degradation of the more labile VapB by Lon protease results in the accumulated level of VapC toxin. Once activated, VapC inhibits mRNA transcription presumably by site-specific cleavage of tRNA^fMet^, which plays a crucial role in the protein synthesis of bacteria (Bunker *et al*. 2008; Winther and Gerdes 2012). HipA function acts in similar manner to VapC, but has different binding sites. In contrast to phosphorylate EF-Tu, free HipA inactivates GltX by phosphorylation at its ATP-binding site Ser^239^, and thus GltX is unable to charge tRNA with glutamate (tRNA^Glu^). Consequently, this induces amino acid starvation and the invariable activation of RelA to more (p)ppGpp synthesis. Thus, high accumulated levels of (p)ppGpp trigger a stringent response that inhibits the global translation process such as protein synthesis (Kaspy *et al*. 2013; Germain *et al*. 2015).

### Targeting DNA gyrase: CcdB and ParE

The *ccd* (couple cell division) locus is adjacent to the origin of replication of the F plasmid and promotes the stable maintenance of F plasmids by coupling host cell division to plasmid proliferation (Ogura and Hiraga 1983). The target of toxin *ccdB*, DNA gyrase, is a ubiquitous bacterial enzyme essential for negative supercoiling of DNA during DNA replication and transcription (Dao-Thi *et al*. 2005; Nöllmann, Crisona and Arimondo 2007). Gyrase is known to consist of two subunits (GyrA and GyrB), GyrA contains a catalytic domain for DNA binding and cleavage, and GyrB contains the ATPase domain. Quinolones are able to inhibit the topoisomerase ligase domain by forming a DNA-topoisomerase-quinolone complex to block DNA and RNA polymerases (Wentzell and Maxwell 2000). The bacterial toxins *ccdB* and *parE* present similar properties to those of quinolones, but interact at a different site to DNA gyrase (Jiang *et al*. 2002; Dao-Thi *et al*. 2005). Under normal growth conditions, the antitoxin CcdA inhibits CcdA toxic activity by forming a tight CcdA_2_–(CcdB)_2_ complex. Once the bacterium loses its plasmid, unstable CcdA degrades and CcdB and GyrA form a symmetric complex. After CcdB*-*GyrA binding, ATP is hydrolysed and the supercoiled DNA is released resulting in blocking bacterial transcription and immediate cell death (Critchlow *et al*. 1997; Dao-Thi *et al*. 2005). More recently, an additional role for CcdB has been that of a persistence factor (Tripathi *et al*. 2012). When faced with antibiotic or heat stress, the increased levels of the ATP-dependent protease Lon (Kuroda *et al*. 2001), responsible for the rapid turnover of unstable proteins, degrade the antitoxin CcdA, freeing toxin CcdB. Free active toxin CcdB causes DNA damage through forming a GyrA-CcdB cleavage complex, which triggers the RecA-mediated SOS response. Ultimately, multidrug-tolerant persister cells are formed.

### Targeting membrane potential: HokB and TisB

**Figure 2.**
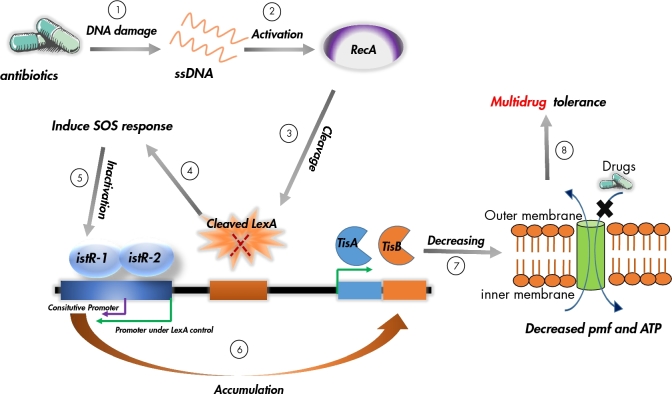
Model of the TisB toxin induced SOS response and persistence formation. (1) Antibiotics (like ciprofloxacin) kill bacteria by damaging their DNA; (2) the SOS response gene *recA* is activated by the accumulation of single-stranded DNA (ssDNA). (3) The induced RecA interacts with the LexA repressor, leading to facilitate the LexA autocleavage. (4) Once the degradation of LexA repressor, the SOS genes are induced to repair DNA damage. (5) Concurrently, the SOS induction results in cleavage of the i*stR-1* pool. (6) The expression of *tisB* is activated by degrading the level of antitoxin IstR-1, this causes membrane damage and the loss of membrane proton motive force (pmf) and ATP level (7); as a result, drugs were drive to out of the cells, leading to persister formation (8). The green and purple arrowheads representing the promoters under LexA control and *istR-1* constitutive promoter, respectively.

*tisB/istR-1* is the first TA locus involved in the SOS response. The locus encodes two small RNA molecules: one is an antisense RNA, *istR-1*, that inhibits TisB toxicity, and the toxin, TisB, under the control of Lex (Vogel *et al*. 2004; Darfeuille *et al*. 2007) and is localised on the inner membrane (Unoson and Wagner 2008) (Fig. 2). The induction of *tisB* results in membrane damage that entails a rapid decrease in DNA replication, RNA transcription and protein synthesis (Unoson and Wagner 2008). HokB is similar to TisB in that both are small proteins, and exert toxicity by damaging the inner membrane. The *hokB-sokB* locus derived from chromosome of *E. coli* K-12 codes for three genes: *sokB*, *mokB* and *hokB* (Pedersen and Gerdes 1999). The *sokB* is a small antisense RNA that controls the translation of the *mokB* reading frame. *hokB* translation is under the control of *mokB*, thereby *sokB* can also suppress *hokB* toxicity. Recent studies have shown that HokB acts as a potential persistence factor (Verstraeten *et al*. 2015) and its accumulated leads to a loss of membrane electrochemical potential, ultimately resulting in persistence.

### Targeting ribosomes: Doc, MazF and RelE

The toxin *doc* (death on cure) and its conjugate antidote, *phd* (prevent host death), are derived from the bacteriophage P1 and play a major role in plasmid stability persistence, programmed cell death and stress response (Lehnherr *et al*. 1993; Gazit and Sauer 1999). Doc has been showed to be a representative member of the Fic protein subfamily, which is ubiquitous in bacteria and involved in crucial functions (such as bacterial pathogenesis) (Garcia-Pino *et al*. 2008; Harms, Maisonneuve and Gerdes 2016). Fic proteins have a central conserved H*X*F*X*(D/E)N(K/G)R motif that is present in Doc structures. Phd dimers are subject to cleavage by CIpXP protease, an ATP-dependent protease of *E. coli* (Lehnherr and Yarmolinsky 1995). It has been shown that mRNA is significantly stabilised upon Doc induction, suggesting that Doc does not cleave mRNA. In fact, Doc toxicity has been proposed to act in a similar manner to hygromycin B (HygB), an aminoglycoside antibiotic (Liu *et al*. 2008). After degradation of Phd by CIpXP protease, the free Doc binds on the 30S ribosomal subunit that includes the HygB-binding site and phosphorylates the conserved threonine (Thr^382^) of the elongation factor EF-Tu. Subsequently, Doc-bound EF-Tu is unable to bind to aminoacylated tRNAs which leads to an accumulation of stalled ribosomes, blocking protein synthesis, and thus a dormant state is formed (Liu *et al*. 2008; Castro-Roa *et al*. 2013). The MazF and RelE proteins are also RNases, which inhibit translation by the cleavage of mRNA. Purified MazF is a sequence-specific (ACA) endoribonuclease, which only cleaves single-stranded mRNA at VUUV' sites independently of the ribosomes, by a mechanism very similar to that of *E. coli* RelE (Christensen *et al*. 2003; Zhang *et al*. 2003; Donegan and Cheung 2009). In the context of the stringent response, antitoxin RelB is degraded by ATP-dependent protease Lon, which leads to activate RelE. The activated RelE induces a global inhibition of translation by cleavage of the mRNA at the ribosome A-site, with the consequence of the tRNA degradation (Christensen and Gerdes 2003; Pedersen *et al*. 2003). Consequently, this activates RelE to trigger a stringent response, creating high-tolerant persisters.

### Targeting bacterial biofilm formation: MqsR

Bacterial biofilms are communities in which cells aggregate on a solid surface and are further enveloped in an exopolysaccharide matrix (Mah and O’Toole 2001; Stewart and Costerton 2001). It has been shown that biofilms are closely linked to antibiotic resistance and that a biofilm can form slimy layers that surround the bacteria and act as a barrier to antimicrobial agents, decreasing the penetration of antibiotics to the bacterium's surface (Davies 2003). When cells are embedded in a biofilm, their MIC has been shown to increase from 6.25 to >400 μg/ml depending on the antimicrobial agent (Evans and Holmes 1987). Besides failure of antibiotic diffusion, some studies have demonstrated that biofilm-associated multidrug-resistant *Pseudomonas aeruginosa* cells can cause slow growth, lipopolysaccharide modification and antibiotic degradation, ultimately accompanied by an increase in antibiotic resistance (de la Fuente-Núñez *et al*. 2013). The first TAs shown to be involved in biofilm formation was *mqsRA*(motility quorum-sensing regulator), a typical type II TAs in which the toxicity of protein MqsR is neutralised by its conjugate antitoxin MqsA (Gonzalez Barrios *et al*. 2005; Brown *et al*. 2009; Wang and Wood 2011). Gonzalez Barrios *et al*. (2005) demonstrated that toxin MqsR is significantly stimulated by biofilm formation and enhanced cell motility. It has been suggested that MqsR is an RNase and prevents translation by cleaving RNAs (Brown *et al*. 2009). In addition, antitoxin MqsA has been linked to the regulation of the general stress responses, such as oxidative stress (Wang *et al*. 2011). Wang *et al*. confirmed that MqsA represses the stress regulator, RpoS, leading to the decreased concentration of messenger 3,5-cyclic diguanylic acid and thus decreasing biofilm formation. However, upon stress, for example, oxidative stress, MsqA is unstable and is rapidly degraded by Lon and ClpXP protease, causing the accumulation of RpoS. As a result, the stringent response is triggered, and the bacterial state is switched from high motility (planktonic) to sessile (biofilm) state.

## BIOLOGICAL ROLE OF TAs IN ANTIMICROBIAL RESISTANCE

Initially, TAs were identified on plasmids and used to be considered as selfish genes with little or no physiological benefit to the host cells. Because if a plasmid encoding the TAs is absent in the daughter cell, the stable toxin is released by rapidly degrading antitoxin to kill plasmid-free cells, in order to increase plasmid maintenance in host cells. Since their discovery, the role of TAs has been debated over decades. Hitherto, mounting evidence has testified that TAs are far more than selfish loci and that they play key roles in promoting cell survival. In particular, in response to antibiotic stress, toxins can be activated by stress-induced protease like CIpXP and Lon. This phenomenon results in slow cellular growth in which the bacterium can now effectively tolerate antibiotic challenge.

## THE MAINTENANCE OF MULTIDRUG RESISTANCE PLASMIDS

Conjugative plasmids identified as reservoirs for resistance genes are one of the most effective physical forums to develop and disseminate the antibiotic resistance genes among bacteria (Carattoli 2013; Mathers, Peirano and Pitout 2015). In many cases, plasmids can carry genes that are highly beneficial to the host bacteria by enabling them to persist in unfavourable environments, e.g. protection against potentially lethal antibiotics. Therefore, plasmids serve as effective DNA shuttles for antibiotic resistance genes that are, in part, linked to the clinical failure of antibiotics treatments. However, because plasmids are extrachromosomal, mobile genetic elements presented in host cells, plasmids impose a metabolic burden to the host cells, which are prone to elimination from bacterial genome in the absence of selective pressure (Zielenkiewicz and Ceglowski 2001). The stable inheritance of plasmids is achieved by activating the plasmid-specified partitioning proteins into dividing cells and selective killing of the cells that failed to acquire a plasmid (Hayes 2003). As discussed above, TAs, like *hok-sok* and *ccdAB*, are responsible for the plasmids stabilisation; thus, TAs also have been viewed as ‘addiction modules’ (Engelberg-Kulka *et al*. 2006). Beside plasmids, TAs appear to play a stabilising role in genomic islands, for instance, SXT, an integrative and conjugative element that mediates tolerance to multiple antibiotics in *Vibrio cholera* (Wozniak and Waldor 2009). One novel TA pair (designated *mosAT*) within SXT has been identified to promote SXT stability. Ectopic expression of *mosT* causes growth inhibition and MosA can neutralise the toxic effect of overexpressed MosT. Similar to plasmid-borne toxins, when SXT is vulnerable to loss, MosT expression is activated to minimise the SXT-free cells. Therefore, the activity of *mosAT* may contribute to the maintenance of SXT in bacterial populations (Wozniak and Waldor 2009).

## BACTERIAL STRESS RESPONSE

The SOS response is important for bacterial survival under stress conditions that can trigger disruption of the DNA replication fork and result in the accumulation of single-stranded DNA. Both RecA and LexA proteins have an important role in the SOS response as regulators (Yamaguchi and Inouye 2011). RecA, activated by single-strand DNA, is involved in the inactivation of the repressor LexA. Normally, LexA binds to a specific sequence in the DNA (the SOS box) and represses the expression of genes involved in DNA repair, mutagenesis and cell growth arrest. The SOS response is an important factor for persister formation in response to the fluoroquinolone antibiotic, ciprofloxacin, which can cause DNA damage (Dorr, Lewis and Vulic 2009; Lewis 2010). The first TA locus, *tisAB-istR-1*, is involved in the SOS response to DNA damage (Vogel *et al*. 2004). This locus encodes a toxic gene *tisAB* and two small RNAs, IstR-1 and IstR-2, as shown in Fig. 2. TisAB is under LexA control and thus activated by the SOS response, but only TisB is responsible to the toxicity (Vogel *et al*. 2004). The transcription of *istR-2* is also SOS regulated and not involved in the TisB control, whereas the antitoxin IstR-1 binds with the LexA-independent promoter and inhibits TisB expression by inducing RNase III-dependent cleavage of *tisB* mRNA (Vogel *et al*. 2004; Darfeuille *et al*. 2007). In the absence of an SOS response, *istR-1* is constitutively transcribed to inactive the toxicity of TisAB by inducing RNase III-dependent cleavage of *tisB* mRNA (Vogel *et al*. 2004). When DNA damage is caused by ciprofloxacin, it activates the RecA protein leading to LexA repressor cleavage, and then the SOS response is induced. The antitoxin IstR-1 that controls the Lex promoter is almost complete cleaved, while the toxin TisB gradually accumulates and rapidly binds to the cytoplasmic membrane, leading to membrane damage, and the proton motive force (pmf) and ATP levels are decreased. This causes the rates of DNA, RNA and protein synthesis to decrease, and the intake of drugs to the cells is blocked. As a result, growth slows down and a multidrug-resistant persister is formed (Vogel *et al*. 2004; Darfeuille *et al*. 2007; Unoson and Wagner 2008; Dorr, Vulic and Lewis 2010) (Fig. 2).

## PERSISTER CELLS

**Figure 3.**
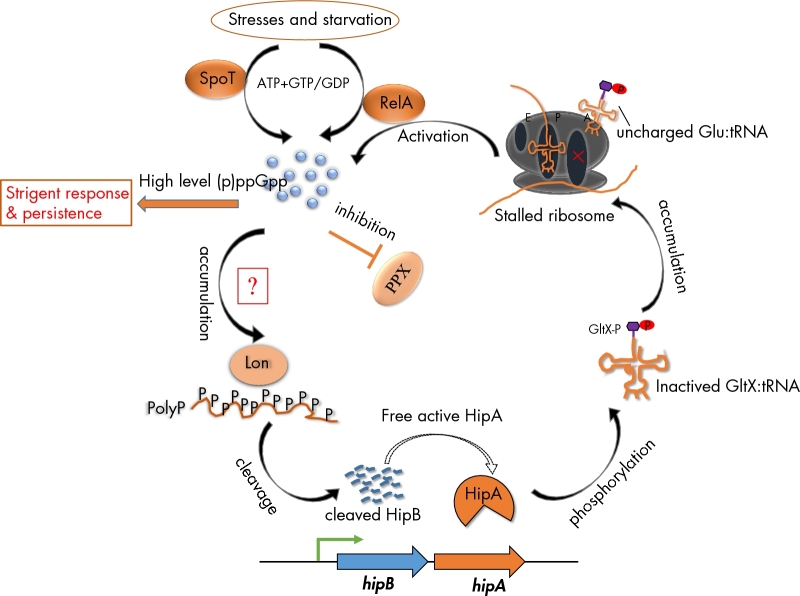
(p)ppGpp-hipA mediated persister pathway. In response to particular stresses, SpoT and RelA are activated to synthesise the nucleotide alarmone (p)ppGpp, The increased (p)ppGpp levels lead to the accumulation of inoganic polyphosphate (PolyP) through inhibition of exopolyphosphatase (PPX), that the cellular enzyme to degrades PolyP. The accumulated PolyP combines with Lon protease preferentially to cleave the antitoxin HipB, resulting in an excess of toxin HipA. In return, free active toxin HipA inactivates GltX by phosphorylation of its ATP-binding site Ser^239^, with the consequence of uncharged tRNA with glutamate (tRNA^Glu^) accumulation in the cell. Uncharged tRNA^Glu^ loads at empty ribosomal sites and triggers the activation of RelA to more (p)ppGpp synthesis, promoting cells entry into dormant state. Note that SpoT and RelA are bifunctional synthetase-hydrolase enzyme, if the stresses have been removed, they can hydrolase (p)ppGpp and bring cells to normal growth (Dalebroux and Swanson 2012). The red box labelled with ‘?’ indicates that the link between stringent response-associated genes (including ppGpp, Lon, PolyP) and TAs has been exploring in some TAs, such as *relBE*, *mazEF* and *yefM-yeoB*. It has been proved that the activation of toxin MazF and YoeB is dependent on the Lon-mediated degradation of their cognates, antitoxins, but not on the accumulation of PolyP and ppGpp (Christensen *et al*. 2001, 2003; Ramisetty *et al*. 2016).

TAs can also contribute to bacteria persistence formation (Lewis 2010; Maisonneuve and Gerdes 2014; Page and Peti 2016). Persistence is observed when a small subpopulation of cells survive antibiotic treatment that has efficiently killed off the rest of the population. In contrast to resistance, persistence is a form of antimicrobial tolerance that is not link with genetic mutation or DNA acquisition, but rather with a spontaneous switch of a dormant, non-dividing state. Therefore, persisters are able to survive in the presence of antibiotics even if they are genetically not programmed to become resistant. More importantly; however, rather than causing cell death, some toxins convert cells into a dormant or a semidormant state that is resistance to antibiotics, and then revive them when environmental conditions become more conducive for growth (Hayes 2003). TAs have been shown to play a major role in persister formation in many model systems. An example of TAs mediating persister states involves the intracellular metabolite, guanosine tetraphosphate and pentaphosphate [(p)ppGpp], the main regulator of the stringent response (Amato, Orman and Brynildsen 2013; Maisonneuve, Castro-Camargo and Gerdes 2013). In *Escherichia coli*, (p)ppGpp was discovered as a alarmone to alter cellular transcription globally by interacting with RNA polymerase activity directly, in response to nutrient starvation or other stress (Dalebroux and Swanson 2012). As a consequence, bacteria can survive even faced with limiting nutrients, suggesting that the coupling accumulation of (p)ppGpp level may induce growth arrest, drug tolerance and the formation of persisters. It has been proposed that high levels of (p)ppGpp trigger persistence by activation of the TA loci, resulting in translation inhibition and growth arrest (Korch, Henderson and Hill 2003; Maisonneuve, Castro-Camargo and Gerdes 2013; Schumacher *et al*. 2015; Harms, Maisonneuve and Gerdes 2016). Contrary to previous reports, there is growing evidence to suggest that EF-Tu is not the target of HipA during the inactivation of translation, but HipA-mediated persistence depends stochastically on the (p)ppGpp-TA pathway (Germain *et al*. 2013; Kaspy *et al*. 2013; Maisonneuve, Castro-Camargo and Gerdes 2013; Wen *et al*. 2014). Most likely, the current molecular model explaining HipA-mediated persistence is shown in Fig. 3 (Korch, Henderson and Hill 2003; Germain *et al*. 2013; Kaspy *et al*. 2013; Maisonneuve, Castro-Camargo and Gerdes 2013; Germain *et al*. 2015). When faced with particular stresses, bacteria rapidly swift transcription profile to trigger the nucleotide alarmone (p)ppGpp synthesis, which involved in catalytic activity of SpoT and RelA, the two (p)ppGpp synthetases of *E. coli* (Dalebroux and Swanson 2012). The resulting increased (p)ppGpp levels accumulate inorganic polyphosphate (PolyP) through inhibition of exopolyphosphatase (PPX), a phosphatase enzyme that degrades PolyP. The accumulation of PolyP combines with Lon protease preferentially to cleave the antitoxin HipB, resulting in an excess of toxin HipA. In return, free active toxin HipA inactivates GltX by phosphorylation of its ATP-binding site Ser^239^, with the consequence of uncharged tRNA accumulation in the cell. Consequently, the amino acid starvation triggers the activation of RelA to more (p)ppGpp synthesis. Thereby, the high level of (p)ppGpp accumulation induces a stringent response that inhibits the synthesis of DNA, RNAs, ribosomal proteins and membrane components, promoting cells entry into dormant state. Conversely, a recent study showed that the activation of *yefM-yoeB* (Christensen *et al*. 2004), a well-characterised type II TAs, is not dependent on the level inorganic PolyP and (p)ppGpp (Ramisetty *et al*. 2016), and further suggested that the pathways of TAs-mediated persistence formation may be far more complicated than previously known.

## CONCLUSION

In the last decade, antimicrobial resistance in Gram-negative pathogens has outpaced the production of novel and even new drugs entering the market place providing an increasing void that is unlikely to be bridged. The drivers and maintenance of antimicrobial resistance was hitherto thought to be antimicrobials themselves; however, increasingly we are becoming aware that antimicrobial resistance is as much to do with genetic maintenance systems, e.g. TAs, as it is to do with the presence of the drug. TAs are remarkable systems that parasitise the bacteria and hold it hostage. TAs are also extremely varied and are a testimony to the dexterity and plasticity of genetic systems to adapt and evolve. Although yet to be fully established, TAs are becoming increasingly numerous and more associated with antimicrobial genes present on the same plasmid thereby providing maintenance of the antimicrobial resistance in the absence of the drug. Worryingly, the SOS induction triggered by drugs such as fluoroquinolones activates TA systems such as TisAB via LexA. The fact that fluoroquinolones are widespread and poorly degraded implies an ever-present pressure on certain TA systems to further be mobilised throughout bacterial populations.

## FUNDING

QY is funded by a CSC scholarship and TRW funded by HEFC. TRW and QY were also supported by MRC grant DETER-XDR-CHINA (MR/P007295/1).

***Conflict of interest*.** None declared.
